# Supplementary material for: Semiquantitative Real-Time PCR to Distinguish Pneumocystis Pneumonia from Colonization in a Heterogeneous Population of HIV-Negative Immunocompromised Patients
Source: Microbiol Spectr. 2021 Aug 4;9(1):10.1128/spectrum.00026-21. doi: 10.1128/spectrum.00026-21 (PMC8552647; doi:10.1128/spectrum.00026-21)
Supplement: SUPPLEMENTAL FILE 1 — Supplemental material. Download SPECTRUM00026-21_Supp_1_seq1.pdf, PDF file, 0.7 MB [file spectrum00026-21_supp_1_seq1.pdf]

Semiquantitative real-time PCR to distinguish *Pneumocystis* pneumonia from colonization in a heterogenous population of HIV-negative immunocompromised patients

Supplementary data (Five tables, eight figures)

Stine Grønseth,[a]# Tormod Rogne,[b,i], Raisa Hannula,[c] Bjørn Olav Åsvold,[d,e,f] Jan Egil Afset,[a,g] Jan Kristian Damås[a,c,h]

[a]Department of Clinical and Molecular Medicine, NTNU, Trondheim, Norway

[b]Department of Circulation and Medical Imaging, NTNU, Trondheim Norway

[c]Department of Infectious Diseases, St. Olavs hospital, Trondheim University Hospital, Norway

[d]K.G. Jebsen Center for Genetic Epidemiology, Department of Public Health and Nursing, NTNU, Trondheim, Norway

[e]HUNT Research Center, Department of Public Health and Nursing, NTNU, Levanger, Norway

[f]Department of Endocrinology, St. Olavs hospital, Trondheim University Hospital, Norway

[g]Department of Medical Microbiology, St. Olavs hospital, Trondheim University Hospital, Norway

[h]Centre of Molecular Inflammation Research, NTNU, Trondheim, Norway

[i]Department of Chronic Disease Epidemiology and Center for Perinatal, Pediatric and Environmental Epidemiology, Yale School of Public Health, New Haven, CT, USA

Running title: Semiquantitative real-time PCR to diagnose non-HIV PCP

#Corresponding author:

Stine Grønseth

Department of Clinical and Molecular Medicine, Faculty of Medicine and Health Sciences

NTNU - Norwegian University of Science and Technology, Trondheim.

Postal address: NTNU Department of Clinical and Molecular Medicine, NO-7491 Trondheim, Norway; Phone: +47-93409532; E-mail: stine.gronseth@ntnu.no

Supplemental Table S1. Independent variables and covariates included in multivariable analyses reported in Table 3

Confounders identified *a priori* (Covariates)

**Independent variables** (Risk factors for PCP)	Age and sex	CCI with and without sex	Comorbidities other than cardiovascular with and without sex	Immunosuppressive condition	Immunosuppressive regimen at presentation	Systemic corticosteroid exposure 60 days preceding presentation	Immunosuppressive regimen last five years (any vs. none)	Daily methylprednisolone equivalent dose at presentation/mg increase	Daily methylprednisolone equivalent dose in mg at presentation among exposed[a]	Cardiovascular comorbidity	Cardiopulmonary comorbidity	Co-infections (antimicrobials other than anti-PCP as surrogate)	Smoking	Lymphocyte count in blood	C_T value of BAL-fluid or tracheal aspirate from semiquantitative real-time PCR-analysis for *P. jirovecii* (i.e.. fungal load)
**Cardiovascular comorbidity**	**X**		**X**	X	X			X	**X**				X		
**C_T value of BAL-fluid or tracheal aspirate from semiquantitative real-time PCR-analysis for P. jirovecii (i.e., fungal load)**	X	X		X	X			X	**X**	X			X	X	
**Immunosuppressive condition**	X														
**Daily methylprednisolone equivalent dose in mg per day at presentation among exposed[a]**	X	X		X						X	X				
**Methylprednisolone equivalent dose in mg per day at presentation**	X	X		X						X	X				
**Dyspnea**	X	X		**X**	X	X	**X**			**X**	X	X			
**Fever**	X	X		X	X	X		**X**	**X**			X			
**At least two cardinal symptoms**	X	X		**X**	X	X	X	**X**	**X**	X	X	X	X		
**All three symptoms**	X	X		X	X	X	X	**X**	**X**	X	X	X	X		
**Abnormal lung auscultation**	X	X		X	**X**			**X**	**X**	X	X				
**Oxygen saturation in %**	X	X						X	X		X	X	X		
**Lymphocyte count x 10^9/L**	X	X		**X**	X	X	X	X	**X**						
**Lymphopenia (< 1.0 x 10^9 cells/L)**	X	**X**		X	X	X	X	**X**	**X**						
**C-reactive protein in mg/L**	X	X		X		X		X	X			X			
**Lactase dehydrogenase level in U/L**	X	X		X		X		X	X			X			
**Atelectasis on thoracic CT**	X	X		X	**X**	X	X	X	**X**	X	X	X	X	**X**	X
**Bronchiectasis on thoracic CT**	**X**	X		**X**	**X**	**X**	X	X	**X**	**X**	X	X	X	X	X
**Crazy paving pattern on thoracic CT**	**X**	X		**X**	**X**	X	X	X	**X**	X	X	X	X	**X**	X

[a]Methylprednisolone equivalent dose per day among 117 exposed patients having an intake the day of *P. jirovecii*-detection.

Abbreviations:

BAL, Bronchoalveolar lavage; CT, Computed tomography; C_T, cycle threshold; PCR, polymerase chain reaction.

Supplemental Table S2. Clinical data of 39 PCP⁺-patients [a] with C_T values from semiquantitative real-time PCR-analysis of bronchoalveolar lavage fluid or tracheal aspirate in gray zone (> 36)													
**Patient ID**	**Age** (years)	**Sex**	**CCI**	**Smoking history**	**Respiratory sample and C_T value**	**Result DIF**	**Immunosuppressive condition**	**Immunosuppression at presentation**	**Corticosteroid exposure and dose at presentation** (mg/day)[b]	**Cardinal symptoms**	**Oxygen saturation** (%)[c]	**Neutrophil count** (x 10^9/L)	**Lymphocyte count** (x 10^9/L)
2	64	F	8	Yes	BAL-fluid; 37	(.)	Solid tumor (genitourinary tract)	None	Intermittent; 0	Dyspnea, fever	94 (-)	2.4	(.)
7	72	F	4	No	BAL-fluid; > 40	Positive	Rheumatoid arthritis	sDMARDs in monotherapy	None	Dyspnea, fever	83 (-)	(.)	(.)
8	82	M	7	No	BAL-fluid; 37	Negative	Chronic lymphatic leukemia with AIHA/ITP	Chemotherapy and steroids	Intermittent; 0	Dyspnea	90 (-)	(.)	(.)
15	79	M	9	No	BAL-fluid; > 40	(.)	Solid tumor (genitourinary tract with pulmonary metastasis)	Chemotherapy	None	All three	93 (-)	3.5	(.)
22	69	F	6	Yes	BAL-fluid; 37	(.)	Non-Hodgkin lymphoma	Steroids in monotherapy	Intermittent; 8	Dyspnea, fever	75 (-)	17.9	(.)
25	67	M	5	Yes	BAL-fluid; 37	Positive	Non-Hodgkin lymphoma	Chemotherapy and steroids	(.); 16	Cough	(.)	17.0	2.8
30	83	M	5	No	BAL-fluid; 37	(.)	Rheumatoid arthritis	sDMARDs in monotherapy	None	Dyspnea, fever	90 (+)	16.3	0.9
36	79	F	6	No	BAL-fluid; > 40	Negative	Suspected autoimmune disease with AIHA and kidney involvement	None	None	Dyspnea	90 (-)	3.9	1.4
48	67	M	8	Yes	BAL-fluid; 38	(.)	Solid tumor (gastrointestinal tractus)	Chemotherapy	None	All three	93 (-)	0.79	(.)
49	83	M	14	Yes	BAL-fluid; 38	(.)	Solid tumor (lungs)	Chemotherapy and steroids	Intermittent; 0	All three	80 (-)	(.)	(.)
62	64	M	7	Yes	BAL-fluid; 37	(.)	Acute myeloblastic leukemia	GVHD prophylaxis or treatment	Daily; 4	Dyspnea	94 (-)	(.)	(.)
69	61	M	4	No	BAL-fluid; 39	Negative	Solid tumor (sarcoma neck region)	Chemotherapy	None	Cough, dyspnea	95 (-)	3.0	(.)
78	72	M	5	Yes	BAL-fluid; 37	Negative	Chronic lymphatic leukemia	None	None	Dyspnea, fever	92 (-)	4.0	0.45
85	55	F	3	No	BAL-fluid; > 40	(.)	Chronic myelogenous leukemia	GVHD prophylaxis or treatment	None	Dyspnea, fever	96 (-)	6.8	1.2
101	65	M	3	Yes	BAL-fluid; 37	(.)	Chronic obstructive pulmonary disease	None	None	All three	(.)	(.)	(.)
103	56	F	7	Yes	BAL-fluid; 39	(.)	Solid tumor (lungs)	Chemotherapy and steroids	Daily; 20	All three	96 (-)	8.3	0.66
109	72	M	5	No	BAL-fluid; 38	(.)	Non-Hodgkin lymphoma	Chemotherapy and steroids	Daily; 32	All three	(.)	9.4	(.)
117	82	F	6	No	BAL-fluid; 37	(.)	Non-Hodgkin lymphoma	Chemotherapy and steroids	Intermittent; 0	Dyspnea, fever	90 (-)	4.8	0.41
126	31	F	3	No	BAL-fluid; 37	Positive	Eosinophilic granulomatosis with polyangiitis	Steroids and azathioprine	Daily; 32	Dyspnea	(.)	12.8	1.0
132	33	M	2	No	BAL-fluid; 37	(.)	Hodgkin's lymphoma	Chemotherapy and steroids	Intermittent; 0	All three	98 (-)	11.2	(.)
135	67	M	6	Yes	Tracheal aspirate; > 40	(.)	Interstitial lung disease	None	None	Dyspnea, fever	74 (+)	(.)	(.)
136	64	M	5	Yes	BAL-fluid; 37	(.)	Solid organ transplant (kidney)	Graft rejection prophylaxis	Daily; 4	All three	96 (-)	(.)	(.)

143	66	M	4	No	BAL-fluid, 37	Positive	Non-Hodgkin lymphoma	Chemotherapy	None	All three	98 (-)	2.0	(.)
145	60	F	4	Yes	BAL-fluid; 37	(.)	Acute lymphoblastic leukemia	Chemotherapy and steroids	Intermittent; 0	Cough, fever	(.)	2.9	(.)
156	74	F	9	No	BAL-fluid; 37	(.)	Solid tumor (breast)	None	None	Dyspnea, fever	87 (-)	(.)	(.)
162	59	M	2	No	BAL-fluid; 37	(.)	Rheumatoid arthritis	sDMARDs and steroids	Intermittent; 0	All three	94 (-)	6.2	2.1
176	41	F	2	(.)	BAL-fluid; 37	Negative	Hodgkin's lymphoma	Chemotherapy	None	All three	93 (-)	1.5	(.)
194	76	M	7	Yes	BAL-fluid; 37	(.)	Chronic lymphatic leukemia	None	None	All three	81 (-)	2.4	1.6
203	77	M	6	Yes	BAL-fluid; 38	(.)	Autoimmune hemolytic anemia	None	Intermittent; 0	Dyspnea, fever	(.)	3.9	2.4
209	82	M	7	Yes	BAL-fluid; 37	(.)	Vasculitis	Steroids in monotherapy	Daily; 32	All three	75 (-)	9.8	0.50
211	62	M	8	Yes	BAL-fluid; 37	(.)	Solid tumor (genitourinary tract)	Chemotherapy and steroids	Intermittent; 0	Dyspnea, fever	92 (+)	1.9	0.60
220	64	M	9	Yes	BAL-fluid; > 40	Negative	Solid tumor (lungs)	Chemotherapy and steroids	Intermittent; 16	All three	78 (+)	9.5	0.20
236	62	F	5	Yes	BAL-fluid; > 40	(.)	Chronic myelogenous leukemia	Chemotherapy	None	Dyspnea, fever	92 (+)	5.7	0.99
245	25	F	2	No	BAL-fluid; 38	(.)	Non-Hodgkin lymphoma	Chemotherapy and steroids	None	Cough, dyspnea	100 (-)	2.3	1.1
253	29	F	2	No	BAL-fluid; 37	(.)	Hodgkin's lymphoma	Chemotherapy and steroids	Intermittent; 0	Cough, dyspnea	95 (-)	6.5	1.9
259	81	F	11	Yes	BAL-fluid; > 40	(.)	Multiple myeloma	Chemotherapy and steroids	Intermittent; 0	Dyspnea, fever	90 (-)	0.6	1.6
262	74	F	4	Yes	BAL-fluid; 40	(.)	Ulcerative colitis	None	None	Cough	(.)	8.2	1.4
282	76	F	5	No	BAL-fluid; 38	Negative	Non-Hodgkin lymphoma	Chemotherapy and steroids	Intermittent; 0	Cough, fever	(.)	2.3	0.30
284	73	F	5	Yes	BAL-fluid; 38	(.)	Anti-synthetase syndrome	Steroids in monotherapy	Intermittent; 0	Dyspnea, fever	85 (+)	15.6	1.1

[a]Criteria for PCP were multimodal and based on available patient data (See Methods and Supplemental Figure S1). Patients not fulfilling the criteria for their respective groups were considered colonized with *P. jirovecii* (i.e., PCP$^-$)

[b]Systemic corticosteroid exposure 60 days preceding presentation and methylprednisolone equivalent dose in mg/day at presentation

[c]Oxygen saturation was measured with (+) or without (-) supplemental oxygen.

Abbreviations and notations:

AIHA, Autoimmune hemolytic anemia; BAL, bronchoalveolar lavage; CCI, Charlson Comorbidity index; DIF, direct immunofluorescence; F, female; GVHD, Graft *versus* host disease; ID, identification number; ITP, Immunologic thrombocytopenic purpura; M; male; PCP, *Pneumocystis* pneumonia; PCR, polymerase chain reaction; sDMARDs, synthetic disease modifying anti-rheumatic drugs; (.) = “missing”.

Supplemental Table S3. Subgroup analyses of 136 PCP^{+}-patients based on semiquantitative real-time PCR-analysis of BAL-fluid or tracheal aspirate[a]

	No. of observations in case of missing (%)	C_T value < 31 No. (%) 22 (16.2)	C_T value $\geq$ 31 No. (%) 114 (83.8)	p-value difference
**Demographics**				
Median age (q_1-q_3)	NA	65 (55-69)	65.5 (58-74)	0.31
Male sex, no. (%)	NA	15 (68.2)	64 (56.1)	0.29
History of smoking, no. (%)	131 (96.3)	12 (54.5)	63 (57.8)	0.78
Median Charlson comorbidity index (q_1-q_3)	NA	4.5 (4-7)	6 (4-8)	0.14
**Immunosuppressive condition**				
Distribution across PCP-groups no. (%)	133 (97.8)[b]			0.050
Hematological malignancies		7 (31.8)	45 (40.5)	
Solid tumors		6 (27.3)	31 (27.9)	
Immunological disorders		1 (4.5)	18 (16.2)	
Solid organ transplantation		8 (36.4)	12 (10.8)	
Chronic lung diseases		0 (0.0)	5 (4.5)	
**Iatrogenic immunosuppression, chemotherapy and corticosteroid exposure at presentation**				
Regimen at presentation, no. (%)	NA			0.059
Chemotherapy for hematological malignancy and adjuvant steroids		4 (18.2)	27 (23.7)	
Chemotherapy for solid tumor and adjuvant steroids		4 (18.2)	11 (9.6)	
Chemotherapy for hematological malignancy		2 (9.1)	5 (4.4)	
Chemotherapy for solid tumor		0 (0)	9 (7.9)	
Corticosteroids in monotherapy		2 (9.1)	14 (12.3)	
Graft rejection prophylaxis after SOT		8 (36.4)	12 (10.5)	
DMARDs with or without adjunctive steroids		1 (4.5)	11 (9.6)	
Other combinations[c]		0 (0)	5 (4.4)	
None		1 (4.5)	20 (17.5)	
Systemic corticosteroid exposure pattern 60 days preceding presentation, no. (%)	134 (98.5)			0.056
Daily		12 (57.1)	41 (36.3)	0.037
Intermittent		7 (33.3)	36 (31.9)	0.13
None		2 (9.5)	36 (31.9)	Ref.
Methylprednisolone equivalent dose in mg/day at presentation, median (q_1-q_3)[d]	134 (98.5)	10 (4-24)	10 (6-20)	0.57

[a]Criteria for PCP were multimodal and based on available patient data (See Methods and Supplemental Figure S1). Patients not fulfilling the criteria for their respective groups were considered colonized with *P. jirovecii* (i.e., PCP^{-})

[b]Three patients had immunosuppressive conditions classified as miscellaneous and were excluded from the comparative analysis.

[c]Other combinations include exposure to other immunosuppressants (mycophenolate, azathioprine, cyclophosphamide, calcineurin- and mTOR-inhibitors, cyclosporine and hydroxychloroquine with or without adjuvant steroids) and one patient receiving both graft rejection prophylaxis for solid organ transplantation and chemotherapy for hematological malignancy with adjuvant corticosteroids

[d]Median methylprednisolone equivalent dose was calculated among 63 patients having an intake the day of *P. jirovecii*-detection.

Abbreviations:

BAL, bronchoalveolar lavage; CT, computed tomography; C_T, cycle threshold; DMARDs, disease modifying anti-rheumatic drugs; NA, not applicable; PCR, polymerase chain reaction; Ref., reference group in logistic regression analysis; SOT, solid organ transplantation.

Supplemental Table S4. Summary of linear regression analysis for immunosuppressive conditions predicting C_T values from semiquantitative real-time PCR-analysis of BAL-fluid or tracheal aspirate for *Pneumocystis jirovecii*-detection[a]

**Model** (167 observations)	**Coefficient**	**Standard Error**	**t**	**P>|t|**	**95 % Confidence interval**
Immunosuppressive condition					
Hematological malignancy	NA	NA	NA	NA	NA
Solid tumor	-0.78	0.75	-1.04	0.30	(-2.26)-0.70
Immunological disorder	1.28	0.88	1.46	0.15	-0.45-3.01
Solid organ transplantation	-1.86	0.9	-2.09	0.038	(-3.60)-(-0.11)
Chronic lung disease	1.50	1.35	1.11	0.27	(-1.17)-4.17
Constant	35.2	0.48	72.5	<0.001	34.2-36.1

[a](F(4,162) = 3.03; p = 0.019, R^2 = 0.07), adjusted R^2 0.05, Root MSE 3.79.

Abbreviations:

BAL, bronchoalveolar lavage; C_T, cycle threshold; NA, PCR, polymerase chain reaction

Supplemental Table S5. Validity of semiquantitative real-time PCR-analysis of BAL-fluid or tracheal aspiration for discrimination between *Pneumocystis* pneumonia and colonization across immunosuppressive conditions in ROC analyses.

Immunosuppressive condition (no. of patients)	Observations, no. (%)[a]	AUC (95 % confidence interval)	p-value[c]
Hematological malignancy (89)	61 (68.5)	0.82 (0.66-0.98)	0.002
Solid tumor (68)	44 (64.7)	0.78 (0.63-0.92)	0.022
Immunological disorder (38)	27 71.1)	0.72 (0.48-0.97)	0.071
Solid organ transplantation (29)	26 (89.7)	0.94 (0.83-1.00)	0.001
Chronic lung disease (13)	9 (69.2)	0.73 (0.370-1.00)	0.27
Other/miscellaneous[b]	5 (2.1)	NA	NA
Population overall (N = 242)	171[a] (70.7)	0.80 (0.73-0.88)	< 0.001

[a]Missing data were independent of immunosuppressive condition (p = 0.25). Proportion (%) refers to the number of observations within the sub-group of immunosuppressive conditions.

[b]Other/miscellaneous immunosuppressive conditions included four patients with no diagnosed condition, whereas two had received steroids for suspected autoimmune disorder and one patient with statin-induced myositis treated with corticosteroids. Sub-group ROC-analysis was not performed for this group.

[c]The reported p-values corresponds to a null-hypothesis of AUC = 0.5.

Abbreviations:

AUC, area under curve; BAL, Bronchoalveolar lavage; NA, not applicable; PCR, polymerase chain reaction; ROC, receiver operating characteristics.

Supplemental figures

FIGURE LEGENDS

Figure S1. *Pneumocystis* pneumonia case definition for study population.

Based on available data three patient-groups were identified and the following criteria for PCP were applied: *Group 1* i) immunosuppressive state and ii) positive DIF, *Group 2* (characterized by missing or negative DIF microscopy-result) i) immunosuppressive state ii) at least one cardinal symptom of PCP (cough, dyspnea and fever) iii) typical findings on thoracic CT (ground glass opacities and/or infiltrates) and iv) presumptive diagnosis at time of diagnosis; i.e. receiving anti-PCP treatment, *Group 3*: Patients who died in-hospital within 30 days of detection without receiving anti-PCP treatment were evaluated individually with respect to cause of death and PCP-status. Patients not fulfilling the criteria for their respective groups were considered colonized with *P. jirovecii*.

Figure S2. Relationship between microscopic examination and semiquantitative real-time PCR-results. DIF microscopy was performed on 99 of 242 respiratory samples (BAL-fluid (n = 82), sputum (n = 10), induced sputum (n = 4), tracheal aspirate (n = 3). The presence of *Pneumocystis jirovecii* was confirmed in 44 (44.4%) samples. With PCR-analysis as a reference for *P. jirovecii*-detection, the sensitivity of DIF microscopy (i.e., positive examination) was positively associated with low C_T values (i.e., higher fungal loads), regardless of respiratory sample-type (adjusted OR 0.77 95 % CI 0.66-0.89, $p < 0.001$) (*). (**Supplemental Figure S2**).

BAL, bronchoalveolar lavage; C_T, cycle threshold; DIF, direct immunofluorescence; PCR, polymerase chain reaction.

Figure S3. Distribution of semiquantitative real-time PCR-results according to PCP-status. Retrospectively 196 patients were diagnosed with PCP (i.e., PCP^+) while 46 were presumed colonized (i.e., PCP^-). C_T values from semiquantitative real-time PCR-analysis of BAL-fluid or tracheal aspirate overlapped but were significantly lower (i.e., higher fungal loads) among PCP^+-patients ($p < 0.01$) (*). Median C_T value for the population overall was 36 (red horizontal line).

BAL, bronchoalveolar lavage; C_T, cycle threshold; PCP, *Pneumocystis* pneumonia; PCR, polymerase chain reaction.

Figure S4. Distribution of semiquantitative real-time PCR-results according to PCP-status. Retrospectively 196 patients were diagnosed with PCP (i.e., PCP^+) while 46 were presumed colonized (i.e., PCP^-). C_T values of BAL-fluid or tracheal aspirate overlapped though PCP^+-patients had a significantly lower median (i.e., higher fungal loads) (35 *vs.* 38, $p < 0.01$).

BAL, bronchoalveolar lavage; C_T, cycle threshold; PCP, *Pneumocystis* pneumonia; PCR, polymerase chain reaction.

Figure S5. Validity of semiquantitative real-time PCR for differentiation between *Pneumocystis* pneumonia and colonization. Sensitivity (blue line), specificity (orange line) and percentage correctly classified (green line) according to various C_T values as clinical cut-offs for differentiation between PCP and colonization based on 171 observations from semiquantitative real-time PCR-analysis of BAL-fluid or tracheal aspirate.

C_T, cycle threshold; PCP, *Pneumocystis* pneumonia; PCR, polymerase chain reaction.

Figure S6. Relationship between semiquantitative real-time PCR-results, corticosteroid dose and corticosteroid exposure pattern. Methylprednisolone equivalent dose (mg/day) at presentation and exposure pattern the preceding 60 days according to C_T values of BAL-fluid or tracheal aspirate based on 169 observations. The distribution of corticosteroid doses according to C_T values was non-linear. Median C_T value of BAL-fluid or tracheal aspirate was 36 (red horizontal line).

BAL, bronchoalveolar lavage; C_T, cycle threshold; *P. jirovecii*, *Pneumocystis jirovecii;* PCR, polymerase chain reaction.

Figure S7. Relationship between semiquantitative real-time PCR-results and immunosuppressive regimen. Immunosuppressive regimen at presentation according to C_T value of BAL-fluid or tracheal aspirate based on 171 observations.

BAL, bronchoalveolar lavage; Chemoth., chemotherapy; C_T, cycle threshold; hem, hematological; *P. jirovecii*, *Pneumocystis jirovecii;* PCR, polymerase chain reaction; SOT, solid organ transplantation.

Figure S8A-C. ROC-curves of semiquantitative real-time PCR-results of BAL-fluid or tracheal aspirate for discrimination between *Pneumocystis* pneumonia and colonization. **A)** ROC-curve for patients with solid tumors based on 44 samples; **B)** ROC-curve for patients with immunological disorders based on 27 samples; **C)** ROC-curve for patients with chronic lung diseases based on 9 samples.

BAL, bronchoalveolar lavage; C_T, cycle threshold; PCR, polymerase chain reaction, ROC, receiver operating characteristics.

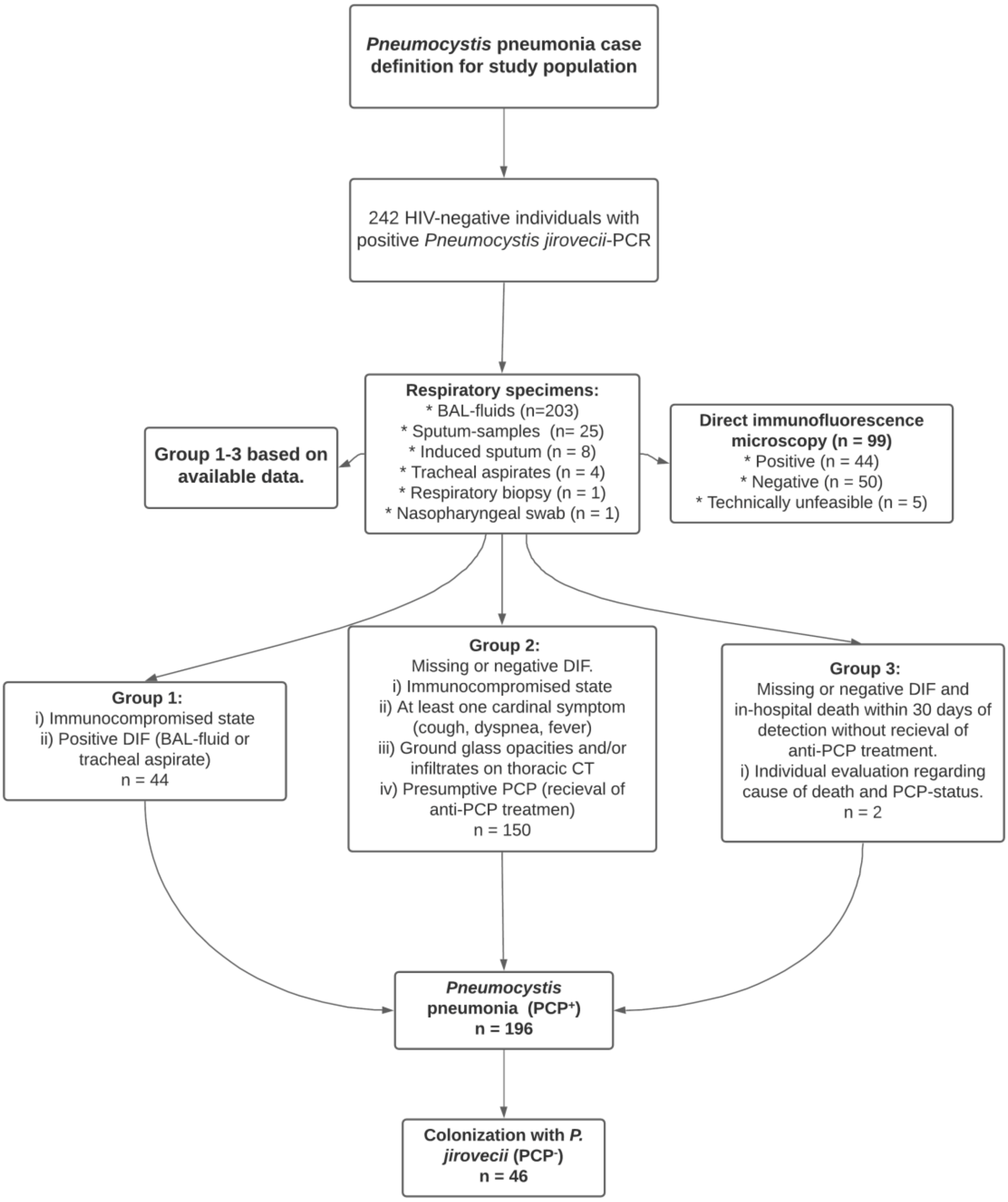

Pneumocystis pneumonia case definition for study population
242 HIV-negative individuals with positive Pneumocystis jirovecii-PCR
Respiratory specimens:
* BAL-fluids (n=203)
* Sputum-samples (n= 25)
* Induced sputum (n = 8)
* Tracheal aspirates (n = 4)
* Respiratory biopsy (n = 1)
* Nasopharyngeal swab (n = 1)
Group 1-3 based on available data.
Direct immunofluorescence microscopy (n = 99)
* Positive (n = 44)
* Negative (n = 50)
* Technically unfeasible (n = 5)
Group 1:
i) Immunocompromised state
ii) Positive DIF (BAL-fluid or tracheal aspirate)
n = 44
Group 2:
Missing or negative DIF.
i) Immunocompromised state
ii) At least one cardinal symptom (cough, dyspnea, fever)
iii) Ground glass opacities and/or infiltrates on thoracic CT
iv) Presumptive PCP (recieval of anti-PCP treatmen)
n = 150
Group 3:
Missing or negative DIF and in-hospital death within 30 days of detection without recieval of anti-PCP treatment.
i) Individual evaluation regarding cause of death and PCP-status.
n = 2
Pneumocystis pneumonia (PCP+)
n = 196
Colonization with P. jirovecii (PCP-)
n = 46

Figure S1

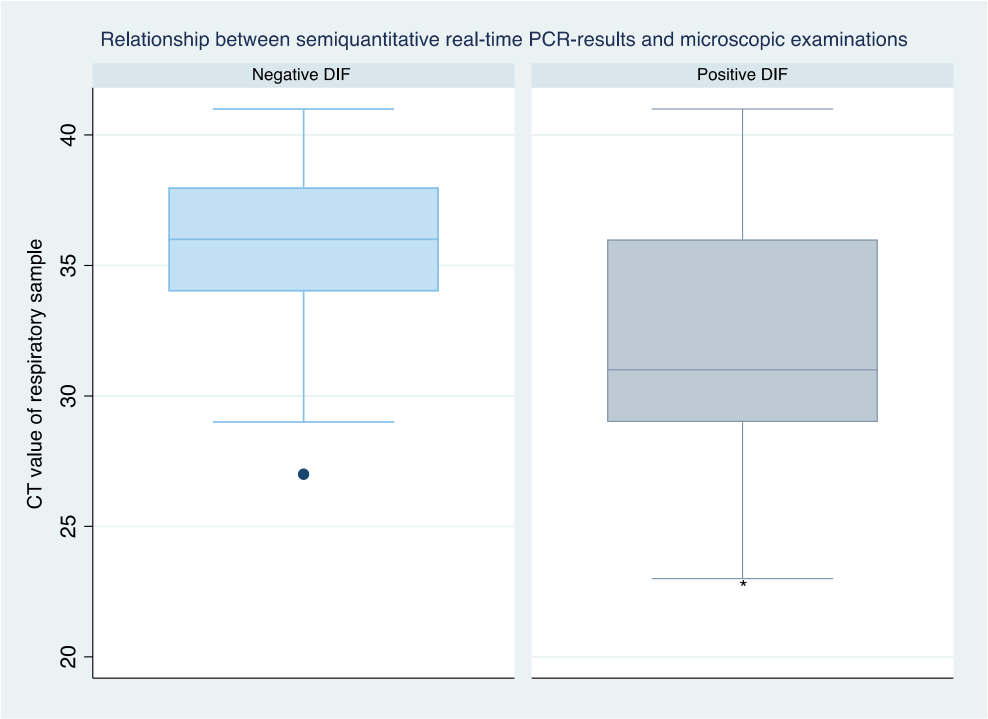

Figure S2

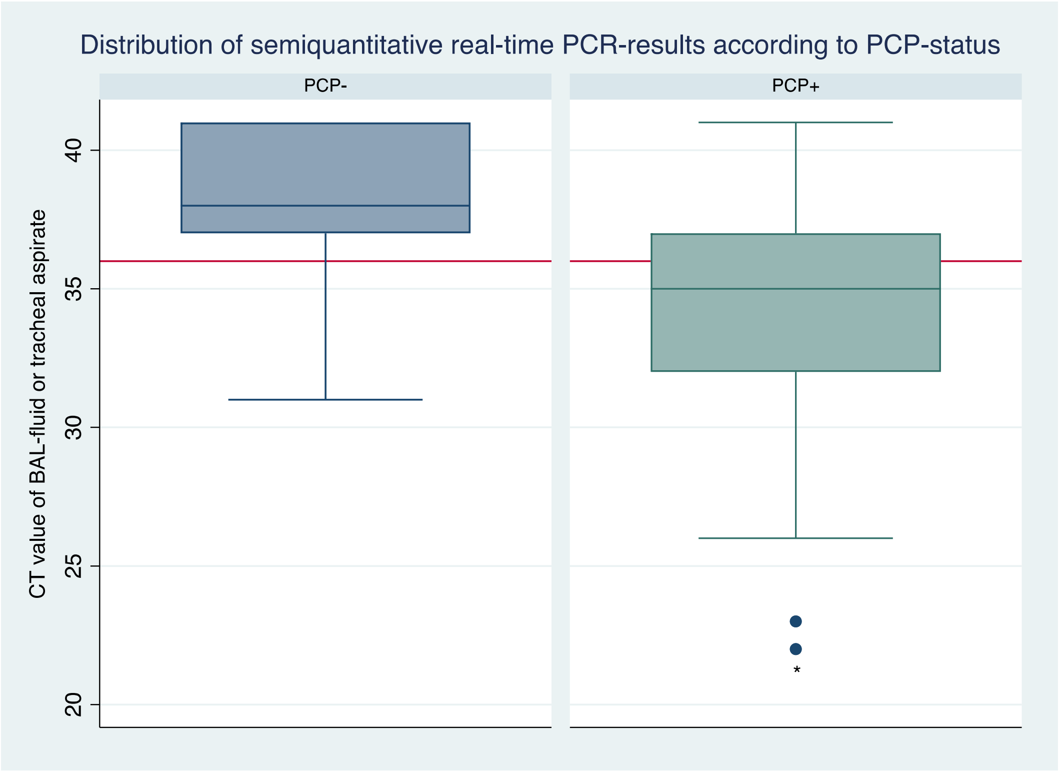

Figure S3

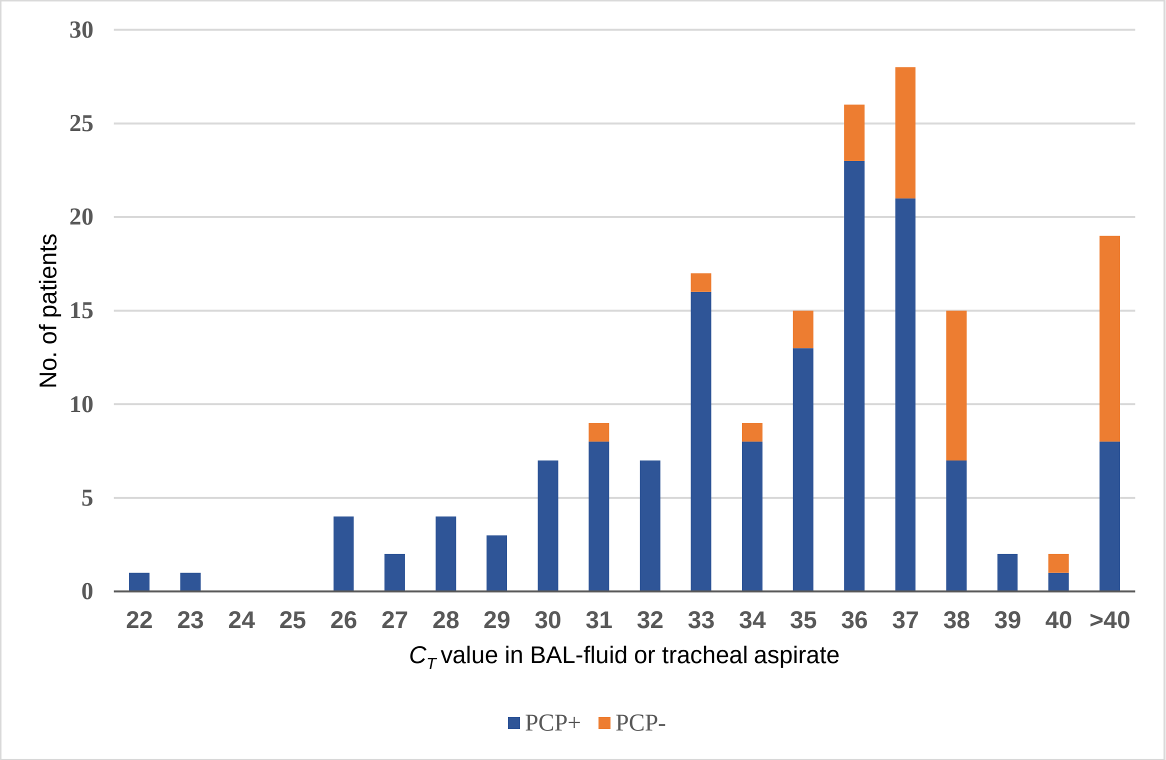

Figure S4

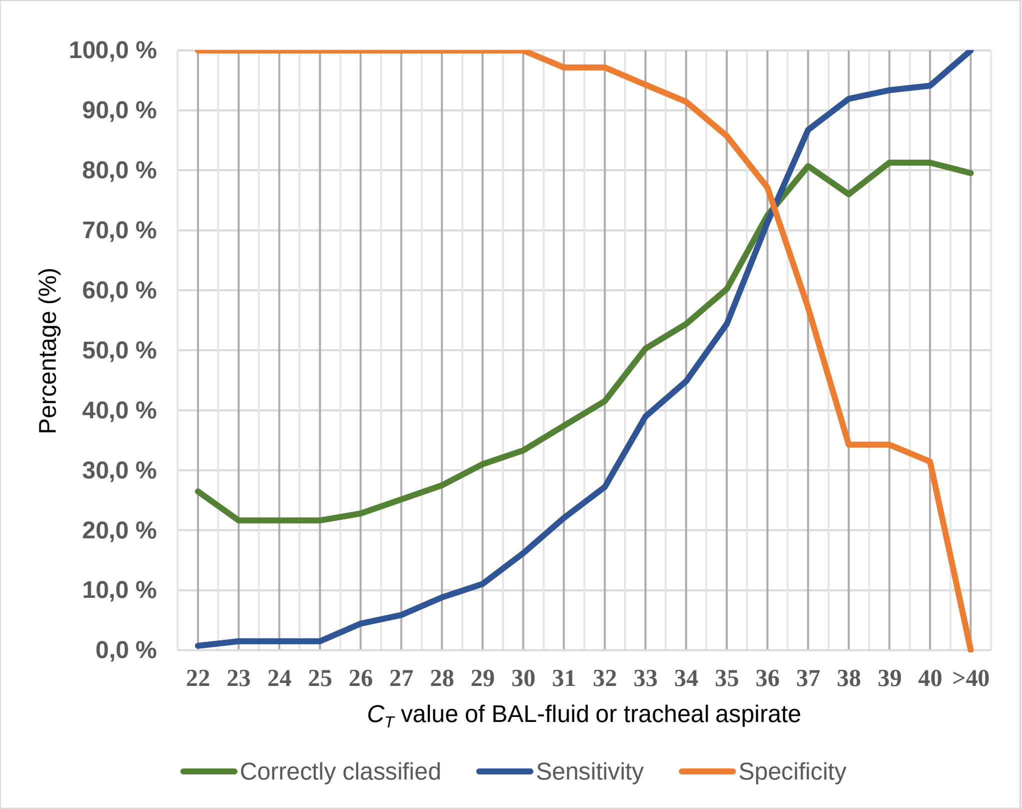

Figure S5

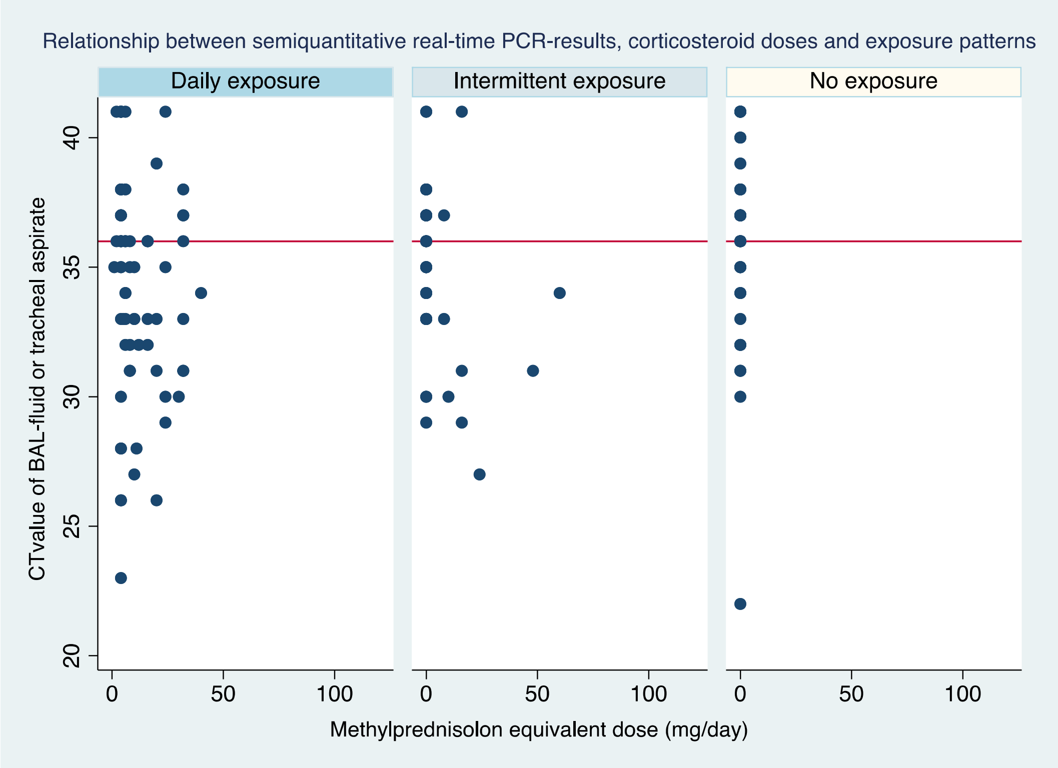

Figure S6

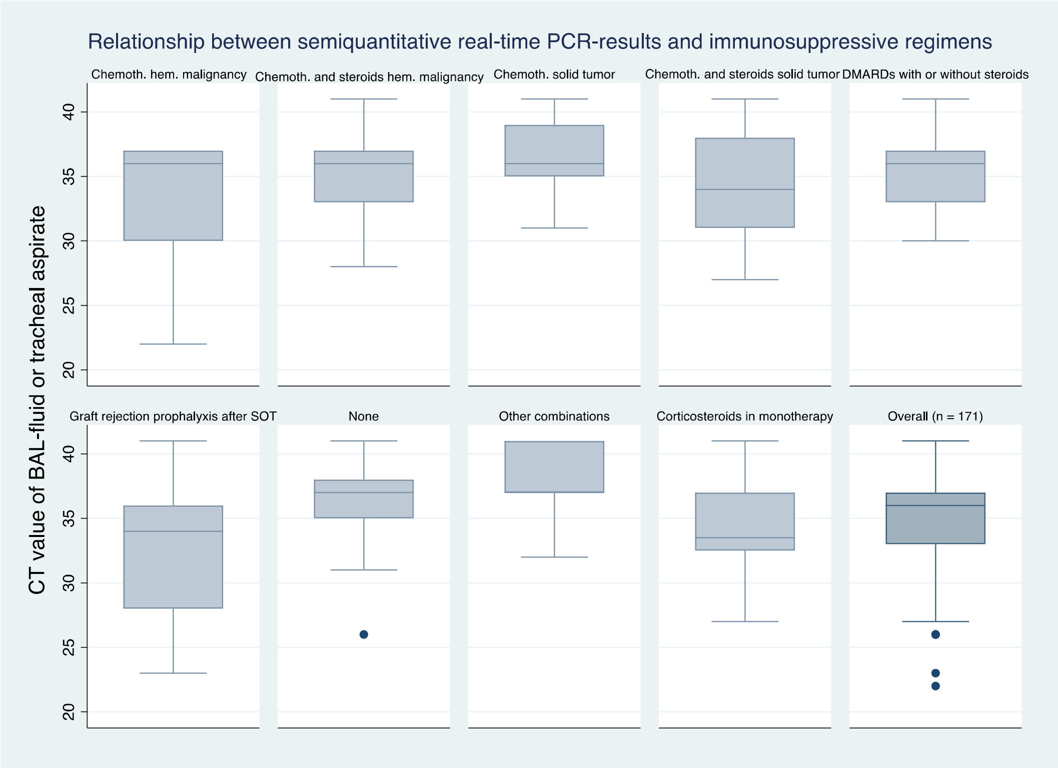

Figure S7

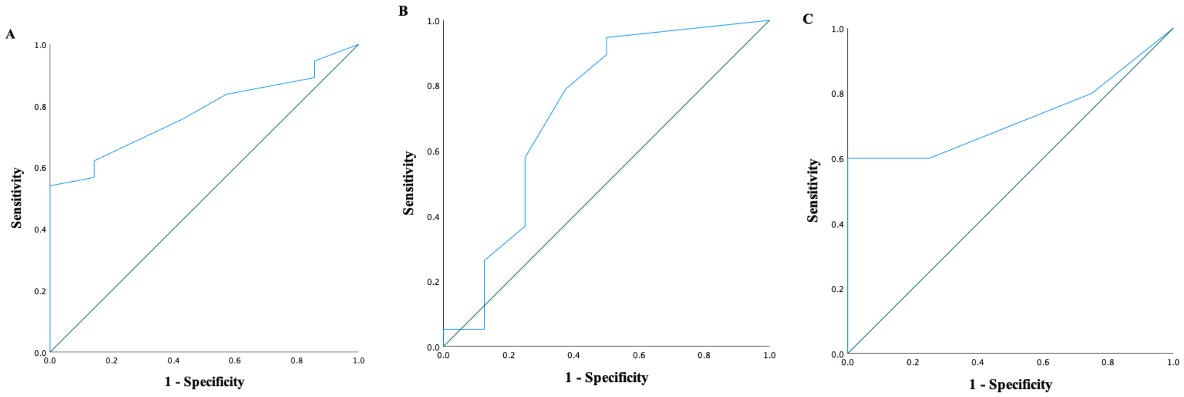

Figure S8A-C
